# Evaluation of Potential Furin Protease Inhibitory Properties of Pioglitazone, Rosiglitazone, and Pirfenidone: An In Silico Docking and Molecular Dynamics Simulation Approach

**DOI:** 10.3390/cimb46080511

**Published:** 2024-08-08

**Authors:** Ahtziri Socorro Carranza-Aranda, Carlos Daniel Diaz-Palomera, Eduardo Lepe-Reynoso, Anne Santerre, José Francisco Muñoz-Valle, Oliver Viera-Segura

**Affiliations:** 1 Doctorado en Ciencias Biomédicas, Centro Universitario de Ciencias de la Salud, Universidad de Guadalajara, Guadalajara 44340, Jalisco, Mexico; ahtziricarranza19@gmail.com; 2 Instituto de Investigación en Ciencias Biomédicas, Centro Universitario de Ciencias de la Salud, Universidad de Guadalajara, Guadalajara 44340, Jalisco, Mexico; daniel.qfb.farma@gmail.com (C.D.D.-P.); eduardo121194@gmail.com (E.L.-R.); biologiamolecular@hotmail.com (J.F.M.-V.); 3 Departamento de Biología Celular y Molecular, Centro Universitario de Ciencias Biológicas y Agropecuarias, Universidad de Guadalajara, Zapopan 45221, Jalisco, Mexico; anne.santerre@academicos.udg.mx

**Keywords:** furin, rosiglitazone, pioglitazone, pirfenidone, molecular docking, molecular dynamics, thiazolidinediones

## Abstract

Furin (Fur) is a member of the protease convertase family; its expression is crucial for cleaving and maturing many proteins. Fur also represents a therapeutic target in cancer, autoimmune diseases, and viral infections. Pioglitazone (PGZ) and rosiglitazone (RGZ) are thiazolidinediones prescribed to type 2 diabetes patients and are structurally similar to the known Fur inhibitors naphthofluorescein (NPF) and pirfenidone (PFD). Thus, this study used molecular docking and molecular dynamics to assess and compare the affinities and the molecular interactions of these four ligands with the Fur active site (FurAct) and the recently described Fur allosteric site (FurAll). The 7QXZ Fur structure was used for molecular dockings, and for the best pose complexes, molecular dynamics were run for 100 ns. The best affinities of the ligand/FurAct and ligand/FurAll complexes were with NPF, PGZ, and RGZ, while PFD presented the lowest affinity. Asp154 was the central residue involved in FurAct complex formation, while Glu488 and Asn310 were the central residues involved in FurAll complex formation. This study shows the potential of RGZ, PGZ, and PFD as Fur competitive (FurAct) and non-competitive (FurAll) inhibitors. Therefore, they are candidates for repurposing in response to future emerging diseases through the modulation of Fur activity.

## 1. Introduction

Secretion protein processing is crucial in developing and functioning higher organism proteins [1,2]. Many of these proteins are synthesized as inactive precursors; therefore, proteolytic cleavage is a critical step for bioactive protein production [3]. Proprotein/prohormone convertase (PC) is one of the most important groups of endoproteolytic processing proteins in mammals and consists of enzymes that exhibit a catalytic subtilisin-like serine protease domain [4,5]. The PC family comprises nine members: PC1/3, PC2, PC4, PC5/6, PACE4, PC7, SKI-1/S1P, PCSK9, and furin. Only four enzymes (PC5/6, PACE4, PC7, and furin) are ubiquitously expressed in tissues and are responsible for the majority of protein processing events [6].

One of the most widely studied proteases is furin (Fur), also known as a dibasic-processing enzyme, paired basic amino acid residue-cleaving enzyme (PACE), and protein convertase subtilisin/kexin 3 (PCSK3). This protease comprises 794 amino acids divided into catalytic and control domains, the latter of which is known as the P domain, whose primary function is stabilizing the catalytic domain [5,6,7]. Due to its ubiquitous expression and localization in the trans-Golgi network, cell surface, and endosomes, Fur plays crucial roles in cleaving and maturing many receptor proteins, enzymes, ligands, and growth factors. Specifically, it is involved in processing Notch, Wnt, and fibroblast growth factor receptors (FGFRs), as well as several cytokines and chemokines. It is also important in regulating cell proliferation, differentiation, and migration.

Fur is also implicated in pathological processes such as cancer, autoimmune diseases, and viral infection, affecting mRNA and protein expression in several tissues [8]. The role of Fur in cancer has been extensively studied, showing that it promotes tumorigenesis and increases malignant tumor phenotype in human head and neck cancer cells [9], as well as in breast [10,11], colorectal [12], and ovarian [13] cancers. Fur also plays a role in the immune tolerance of cancer by contributing to the production of extracellular matrix components to the differentiation of cancer-associated fibroblasts, the epithelial–mesenchymal transition, and the Treg phenotype through TGF-β [14,15,16]. In this sense, Fur inhibition leads to decreased tumorigenicity, motility, and invasiveness of cancer cells [17].

Concerning autoimmune diseases, Fur activity is found to be increased in inflammatory conditions; therefore, it is strongly related to the progression of atherosclerosis [18], rheumatoid arthritis [19,20], lupus erythematosus [21], and Sjogren’s syndrome [22].

In addition, Fur is involved in bacterial and viral infections, increasing pathogenicity. During bacterial infections, this protein processes and modifies toxins, such as anthrax, diphtheria, Shiga toxin, pseudomonas exotoxin A, and Clostridium septicum alpha-toxin, enhancing their toxicity [23,24,25]. Fur also plays a critical role in regulating the viral life cycle during viral infection by processing and maturing glycoproteins on the surface of viral particles [8,26,27]. For example, the human papillomavirus (HPV) requires Fur-mediated cleavage of the L2 minor protein for de novo infections. However, this cleavage can also occur independently from Fur during viral assembly within the host cell [27].

Similarly, flaviviruses, including dengue, Zika, Japanese encephalitis, and West Nile viruses, also rely on Fur-mediated cleavage of the pre-membrane protein for maturation and transitioning from inert to infectious viral particles [8,27,28,29]. In the case of the hepatitis B virus (HBV), Fur serves a dual purpose, aiding in the viral life cycle and processing the E-antigen essential for immune tolerance, potentially leading to chronic infections [30,31]. Due to the above reasons, Fur has become a key target for treating multiple pathologies.

In terms of viruses affecting the respiratory system, influenza virus proteins, specifically those containing hemagglutinin types 5, 7, and 9, are susceptible to Fur cleavage, which significantly increases their pathogenicity and infectivity [8,27,32]. In particular, most coronaviruses use Fur to cleave the spike protein (S), one of the most promising targets for therapy against MERS-CoV, SARS-CoV, and SARS-CoV-2. The spike protein cleavage facilitates interaction with ACE2 for entry into the host cell [33,34,35,36]. Thus, recent efforts to find therapeutic drugs for SARS-CoV-2 have led to the evaluation and repurposing of potential Fur inhibitory molecules using in silico and in vitro assays. In this regard, naphthofluorescein (NPF) has been recently reported as a Fur inhibitor useful against SARS-CoV-2 replication [37], while pirfenidone (PFD), an FDA-approved molecule for treating idiopathic pulmonary fibrosis [38], has recently been recommended as a potential treatment for COVID-19, ameliorating the cytokine storm induced by SARS-CoV-2 infection [39]. Previous studies have shown that PFD also induces a modest Fur inhibition (~50%) at relatively high concentrations (5–8 mM) [40]. However, the mechanism of Fur inhibition by NPF or PFD is unknown.

The current emphasis in pharmacological repurposing is focused on molecules identified as effective inhibitors of Fur activity containing imidazole, such as the sulconazole [10], and thiazolidine rings [5,41], such as thiazolidinediones (TZDs), a group of molecules to which pioglitazone (PGZ) and rosiglitazone (RGZ) belong, which are primarily used in treating type 2 diabetes [42], acting as an α-lipoic acid and activating peroxisome proliferator-activated receptor γ (PPARγ), a nuclear transcription factor that controls glucose metabolism and lipid homeostasis [43]. Beyond this well-established role, TZDs exhibit antifibrotic, anti-inflammatory, and antitumoral effects in various tissues [44]. These effects encompass a reduction in TGF-β expression, extracellular matrix deposition, NFkβ activation, TNFα levels, and inhibition of p38 MAPK activation. TZDs have recently been reported for their antiviral potential against SARS-CoV-2 [45,46,47,48,49]. Although these ligands show great promise, their precise mechanisms of action remain incompletely understood. Considering the promising Fur inhibitor activity of the thiazolidine rings of the TZDs, PGZ, and RGZ and well-known Fur inhibitors such as NPF and PFD, this study focused on the in silico analysis of their molecular interactions and affinity with the active and allosteric sites of the Fur protein and assessed their potential for repositioning as Fur inhibitors for future medical interventions.

## 2. Materials and Methods

### 2.1. Molecular Structures

The three-dimensional (3D) structures of the Fur protein were obtained from the Protein Data Bank (PDB) under IDs 7QXZ, 7LCU, 5MIM, and 7O1Y [50,51,52,53]. The potential Fur ligands included four heterocyclic compounds: NPF (a heteropolycyclic molecule), PFD, PGZ, and RGZ (heteromonocyclic molecules). NPF was obtained from the PubChem CID database, while PFD, PGZ, and RGZ structures were retrieved from the ZINC15 database [54] (Table 1). Additionally, the 3-(3,5-dichlorophenyl)-pyridine derivative (3DPP-D) molecule was used as a reference/control during molecular re-dockings. 3DPP-D is a Fur inhibitor and has been previously reported to be co-crystallized with the Fur structure (Figure S1) [50].

### 2.2. Preparation of Protein and Ligand

In a preliminary step, different Fur structures (PDB IDs: 7QZX, 7LCU, 5MIM, and 7O1Y) were selected to determine the optimum structures for the dockings analysis. All structures were subjected to molecular adaptations, such as the removal of water molecules and extra ligands, addition of hydrogen atoms, and determination of AM1-BCC atomic charges with bond charge corrections (BCCs); energy minimization was also performed, and the structures were refined [55]. Likewise, the structures of the four ligands were prepared by adding hydrogen atoms (polar and non-polar) and Gasteiger atomic charges [56]. All these adjustments were performed using the UCSF Chimera v.1.1.6 (University of California, Oakland, CA, USA), while AutoDock Tools v.1.5.6 (Center for Computational Structural Biology, La Jolla, CA, USA) was used for the creation of the pdbqt files [57,58,59,60].

### 2.3. Molecular Docking Analysis

Using redocking, we identified and validated the coordinates of the active site of Fur (FurAct) based on the binding site location of the following co-crystallographic ligands previously reported as highly potent Fur inhibitors: 3DPP-D (PDB ID: 7QXZ), BOS-318 (PDB ID: 7LCU), 2,5-dideoxystreptamine-derived (PDB ID: 5MIM), and guanylhydrazone-based inhibitor 2 (mi307) (PDB ID: 7O1Y) [50,51,52,53]. This strategy allowed us to determine that the 7QXZ Fur structure was optimum and select it for subsequent molecular dockings (Table S1 and Figure S1).

Considering the resulting parameters, 100 run modes of localized molecular docking on FurAct were carried out for the four molecules of interest (NPF, PFD, PGZ, and RGZ) with a box size of 26 Å × 26 Å × 26 Å and a center x = 51.42, y = −32.10, and z = −6.66 using AutoDock Vina v.2.5.4 and AutoDock4 v.4.2.6 (Center for Computational Structural Biology, La Jolla, CA, USA) and a box size of 40 Å × 40 Å × 40 Å and a center x = 49.35, y = 33.95, and z = −4.297 using the Lamarckian genetic algorithm [61,62].

Furthermore, to identify possible interactions of the four evaluated molecules with important allosteric inhibition sites of Fur, we also ran 100 modes of blind molecular docking, i.e., considering the entire Fur structure, and identified a second interaction site that we named FurAll. This site corresponds to a recently described allosteric site of Fur, as discussed below. The calculations were carried out with the Blind Docking Server [63] and confirmed by AutoDock Vina v.2.5.4 (box size of 65 Å × 58 Å × 70.89 Å with a center x = 35.50, y = −38.54, and z = −1.63) and AutoDock4 v.4.2.6 (box size of 126 Å × 126 Å × 126 Å with a center x = 35.73, y = −38.043, and z = 0.156). The complexes with the highest affinity scores during redocking and docking assays were visualized and analyzed for covalent and non-covalent bonds using PyMOL2 v.2.5.4 [64] and Discovery Studio visualizer v.21.1.0.20298 (BIOVIA, San Diego, CA, USA). Contacts between molecules were considered “real” when bonds presented a length lower or equal to 3.3 Å [65]. The results of the blind docking were corroborated using AutoDock Vina v.2.5.4 and AutoDock4 v.4.2.6 (Figure S3, Table S3) [65]).

### 2.4. Molecular Dynamics Simulation

The best ligand–Fur complexes obtained with each of the four molecules under study and FurAct and FurAll were then prepared for molecular dynamics (MD) simulations using the Nanoscale Molecular Dynamics (NAMD) v.2.14 software (University of Illinois at Urbana–Champaign, Champaign, IL, USA) [66]. The force field parameters for Fur were generated with the CHARMM 27 package, and for the ligands, the topology files were obtained from the CHARMM-GUI server [67,68].

**Figure 1:**
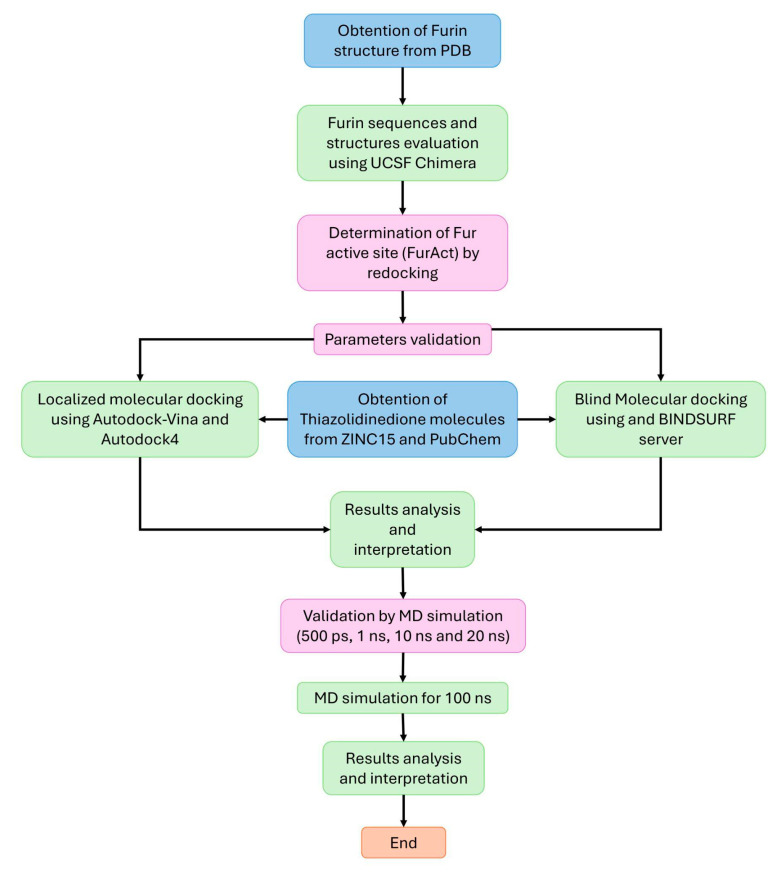
Workflow diagram.

The complete process was performed in five steps according to the NAMD protocols found in https://www.ks.uiuc.edu/Training/Tutorials/ (accessed on 15 September 2023). First, all complexes were prepared by minimizing the structures of the proteins and ligands, eliminating water molecules in 2000 steps. Second, all complexes were solvated in a 3D TIP3P water cube (5 Å dimension), and the periodic boundary conditions presented the following dimensions: x = 75.33 Å, y = 96.17 Å, and z = 87.57 Å for the active site (FurAct) and x = 69.65 Å, y = 65.61 Å, and z = 75.20 Å for the allosteric site (FurAll). Third, all systems were neutralized and minimized by adding sodium, calcium, and chlorine ions. The temperature was set at 310 K and the pressure at 1 atm for two fs for 100 ps. Fourth, all systems were subjected to an equilibrium phase for 200 ps to subsequently run the production dynamics for 100 ns. During the 100 ns of MD generation, the pressure was held at 1 atm by employing the Nose–Hoover–Langevin piston barostat 1.0132 with a Langevin piston decay of 0.1 ps and a period of 0.2 ps. Finally, the MD simulation was analyzed with Carma v.2.01 (Alexandroupolis, Greece) and VMD v.1.9.4 (University of Illinois at Urbana–Champaign, IL, USA) software to examine the root mean square deviation (RMSD), root mean square fluctuation (RMSF), and radius of gyration of amino acids (Rg) through the fluctuations of the furin c-alpha [69,70]. RMSD, Rg, and RMSF are indicators of the element in the MD of protein/ligand complexes and provide us with valuable information to compare the stability of the two protein structures, measure the stability of electrons from their center of gravity, and determine the flexibility of each protein residue during a simulation period. Lower RMSD, Rg, and RMSF values indicate more stability and feasibility of the complex formation [71,72,73]. The graphs were elaborated with RStudio v.4.2.1, and the complexes obtained from MD were compared with those analyzed by molecular docking to corroborate the stability of the interactions; this was conducted using PyMOL2 v.2.5.4 (Schrödinger, New York, NY, USA) [63] and Discovery Studio visualizer v.21.1.0.20298 (BIOVIA, San Diego, CA, USA. Figure 1 shows the workflow diagram for the studied molecules.

## 3. Results

### 3.1. Molecular Docking

**Figure 2:**
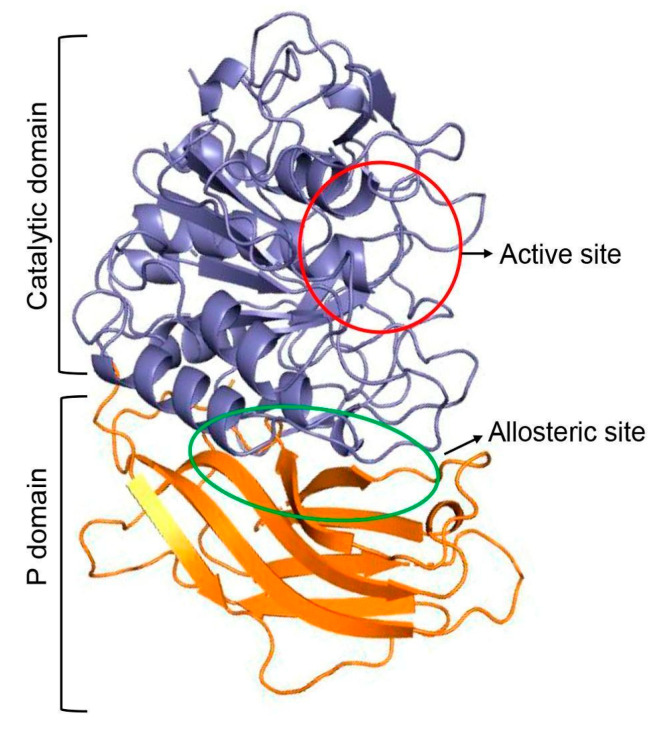
Representation of furin’s 3D general structure and domains (PDB ID: 7XQZ). Figure obtained with PyMOL v.2.5.4.

The Fur structure presents two important regulatory regions: the catalytic domain and the P domain. Within the catalytic domain, the active site (Asp154, His194, Asn295, and Ser368) plays a critical role in the peptidase activity; meanwhile, the P domain (Gly265, Ans310, Gln488, and Ala532) stabilizes the catalytic domain, modulating its activity (Figure 2).

**Figure 3:**
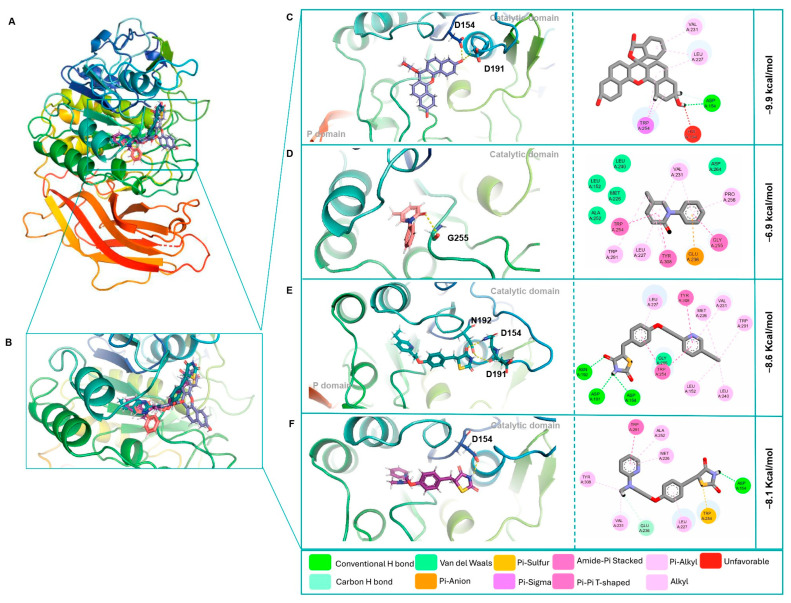
The 3D and 2D representations of the Fur active site (FurAct) and its molecular interactions with the potential ligands. (**A**,**B**) Overview of overlay complexes, (**C**) NPF, (**D**) PFD, (**E**) PGZ, and (**F**) RGZ. Figures obtained with the PyMOL v.2.5.4 and Discovery Studio v.21.1.0.20298 software.

In this study, we assessed the molecular interactions of NPF, PFD, PGZ, and RGZ at both sites and determined their binding affinities as well as the inhibitory potential of Fur’s enzymatic activity. The localized molecular docking assays showed that NPF presented the highest affinity to the Fur active site (FurAct) with a score of −9.9 kcal/mol, followed by PGZ (−8.6 kcal/mol), RGZ (−8.1 kcal/mol), and PFD (−6.9 kcal/mol). Asp154 was the central amino acid residue involved in the ligand/protein complexes with NPF, PGZ, and RGZ. The variation in the affinity score of these three molecules was influenced by the number of polar contacts (1, 3, and 1, respectively) and the distance of the polar bond between Asp154 and its ligands. This distance was longer for NPF (2.7 Å) compared to PGZ (2.6 Å) and RGZ (2.2 Å). On the other hand, the complex formed between FurAct and PFD (−6.9 kcal/mol) presented low affinity and was orchestrated by a single polar bond with Gly255 (Table 2 and Figure 3A–F).

**Figure 4:**
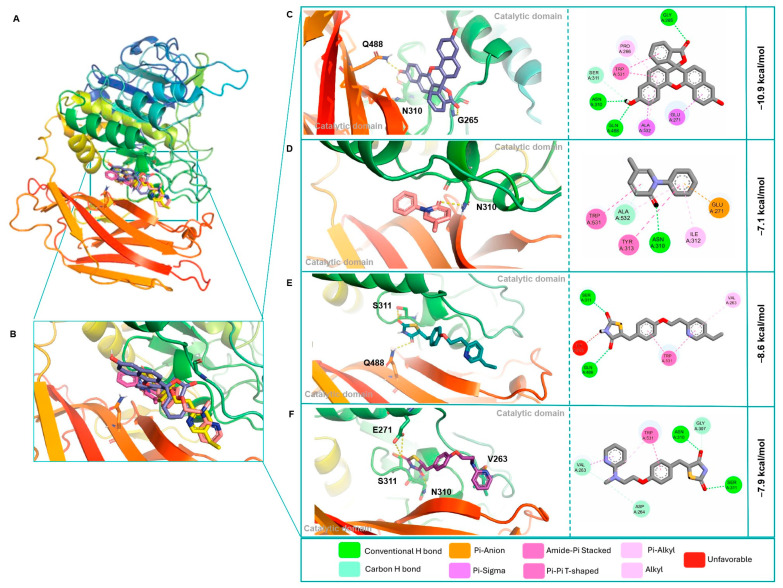
The 3D and 2D representations of the Fur allosteric site (FurAll) and its molecular interactions with the potential ligands. (**A**,**B**) Overview of overlay complexes, (**C**) NPF, (**D**) PFD, (**E**) PGZ, and (**F**) RGZ. Figures obtained with the PyMOL v.2.5.4 and Discovery Studio v.21.1.0.20298 software.

Remarkably, the blind docking with FurAll showed that three of the four ligands presented higher affinity forces for the Fur allosteric than for the Fur active site. NPF showed the highest affinity score (−10.9 kcal/mol), followed by PGZ (−8.7 kcal/mol), RGZ (−7.9 kcal/mol), and PFD (−7.1 kcal/mol). Glu488 and Asn310 were the main amino acid residues involved in the complexes’ formation and stabilization (Table 3 and Figure 4A–F). In all cases, non-covalent interactions, such as electrostatic forces (van der Waals, Pi–cation, and Pi–anion), hydrophobic (Pi and alkyl hydrophobic), and sulfur bonds, favored the stability of the ligand/protein complexes (Table 3, Figure 3A–F and Figure 4A–F).

As for the redocking of the co-crystallized ligand derived from 3DPP-D, it only showed high affinity towards FurAct, where the molecular interactions involved Asp233 (conventional H-bond), Pro256, Asp264, Glu236, and Asp154 (electrostatic interactions) and Trp254, Ala252, Met226, Leu240, Trp291, Val231, and Gly255 (hydrophobic interactions) (Table S2 and Figure S2).

Thus, the molecular docking assays indicated that according to the number of H-bonds, NPF, RGZ, and PFD presented a higher affinity for FurAll than FurAct. In comparison, PGZ showed a higher affinity for the active site, although the binding affinity was more significant with FurAll. The non-polar bindings, such as hydrophobic interactions, probably promoted the increase in affinity to FurAct.

Based on the molecular docking assay using AutoDock4, we obtained the inhibition constant (Ki) of Fur for each of the study molecules. We observed that the theoretical inhibition concentration from the active site was very low for PGZ (1.28 μM), followed by RGZ (3.33 μM) and PFD (24.87 μM); however, for NPF, it could not be calculated. On the other hand, the Ki values towards the allosteric site calculated from blind molecular docking showed a decrease in inhibition concentration for NFP, PGZ, and RGZ. In contrast, for PFD, it could not be calculated due to their preference for FurAct (Table 4).

### 3.2. Molecular Dynamic

To further validate the stability of the ligand/protein complexes, especially those targeting the active and allosteric sites of Fur, MD simulations were conducted, starting with the most optimal conformations identified through molecular docking. The movements of atoms and molecules of Fur alone (ligand-free) in systems (water and ions) were also considered to compare the conformational changes in the presence of molecules.

**Figure 5:**
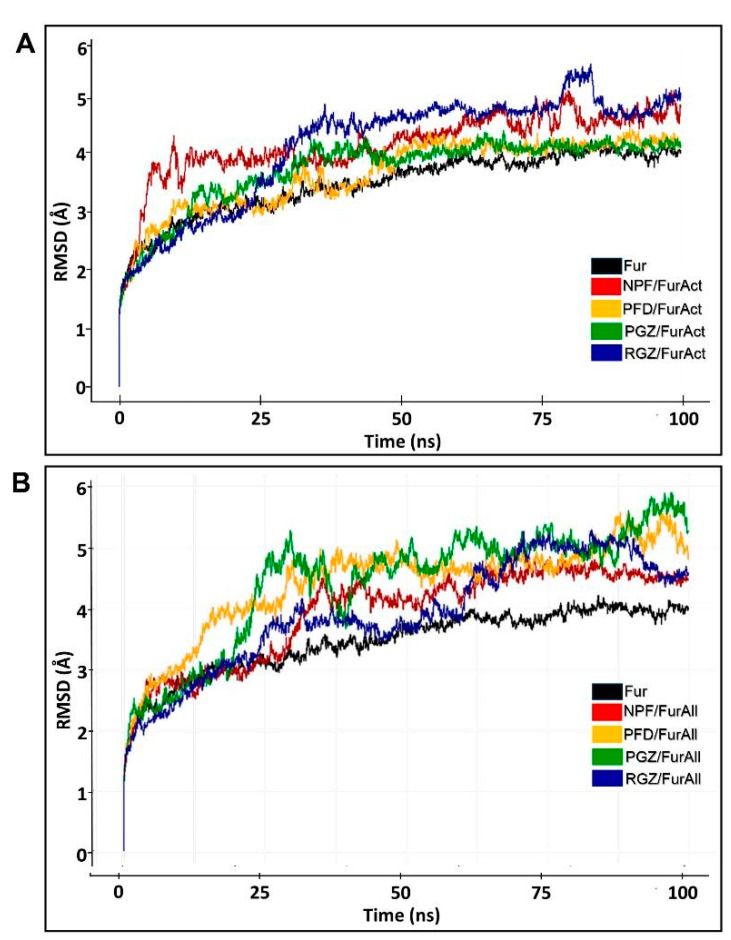
RMSD of ligands/Fur complexes during MD simulation with (**A**) active site and (**B**) allosteric site of Fur. RMSD of complexes of NPF (naphtofluorescein), PFD (pirfenidone), PGZ (pioglitazone), and RGZ (rosiglitazone).

The structural integrity of all complexes was assessed using the root mean square deviation of atomic position value (RMSD). The mean of RMSD of the ligand-free Fur protein (3.98 ± 0.07 Å), PFD/FurAct (4.21 ± 0.08 Å), and PGZ/FurAct (4.12 ± 0.06 Å) complexes were similar, unlike the NPF/FurAct (4.67 ± 0.13 Å) and RGZ/FurAct (4.77 ± 0.14 Å) complexes, which displayed higher RMSD values (Table 5 and Figure 5A). Notably, the interactions of NPF and RGZ with the active site of Fur (FurAct) exhibited greater RMSD fluctuations in the last 10% of the MD simulation.

Among the four analyzed ligands/FurAll complexes, NPF presented the lowest RMSD value (4.52 ± 0.05 Å), followed by PGZ (4.66 ± 0.17 Å), PFD (5.22 ± 0.17 Å), and RGZ (5.57 ± 0.17 Å) (Table 5 and Figure 5B). The RGZ/FurAll, PGZ/FurAll, and PFD/FurAll complexes showed higher RMSD values compared to their RGZ/FurAct, PGZ/FurAct, and PFD/FucAct counterparts, suggesting that they established more stable contacts at the Fur active site than at the Fur allosteric site, probably due to a stabilizing failure at the end of the MD simulations. Contrary to the above, NPF/FurAll established a more stable complex, as supported by the statistical similarity between the RMSD values of these complexes; also, the RMSD values in NPF/FurAll and PGZ/FurAll were similar to those in ligand-free Fur protein (Table 5 and Figure 5B).

Based on this information, we assessed the ligand/protein complexes’ general stability and structural compactness for both FurAct and FurAll using the radius of gyration (Rg) values. For FurAct, we observed a high consistency of Rg values in the union of NPF and PGZ throughout the entire trajectory, compared to those with PFD and RGZ. The Rg value of PFD/FurAct remained similar to those of the ligand-free Fur protein during the entire MD simulation.

**Figure 6:**
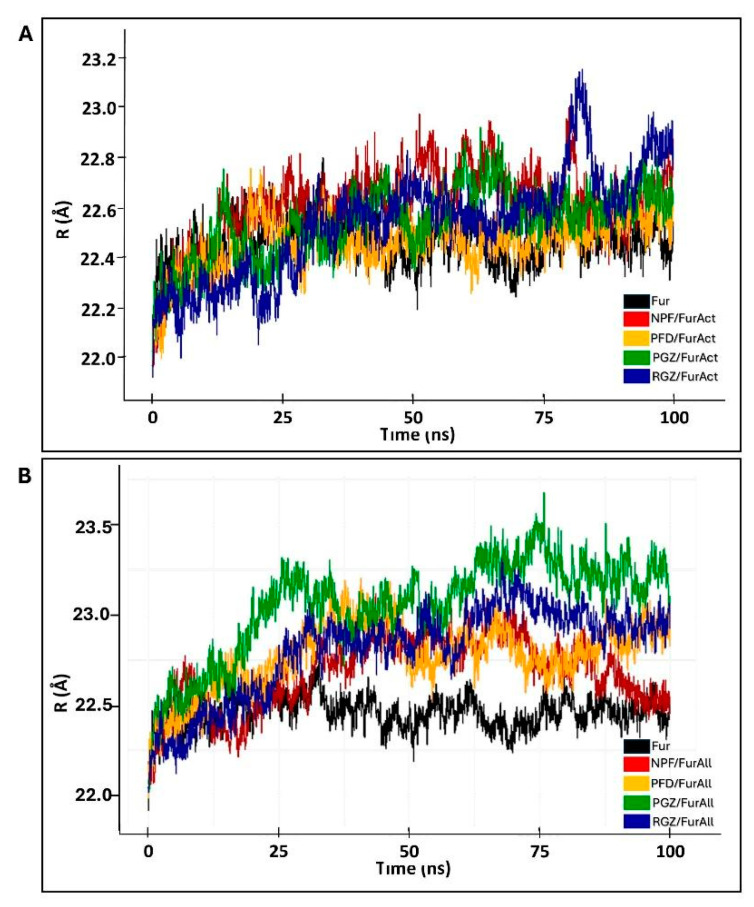
Rg of ligands/Fur complexes during MD simulation with (**A**) active site and (**B**) allosteric site of Fur. Rg of complexes of NPF (naphtofluorescein), PFD (pirfenidone), PGZ (pioglitazone), and RGZ (rosiglitazone).

The degree of compactness of the complexes was similar (lowest Rg values) for FurAll and FurAct, highlighting the NPF/FurAll complex as the most compact one, followed by PFD/FurAll and PGZ/FurAll (Table 6 and Figure 6A,B). Regarding the complex RGZ/FurAll, an increase in complex size was observed in the last 10 ns (Table 6 and Figure 6B).

Another critical parameter that contributes to understanding the dynamics of the ligand/protein complexes during MD simulation is the RMSF value (root mean square fluctuation). This value calculates the flexibility of specific amino acid residues and how they contribute to molecular motion within the complexes. High RMSF values indicate high amino acid mobility, as opposed to low RMSF values, which are associated with secondary protein structures with high stability.

**Figure 7:**
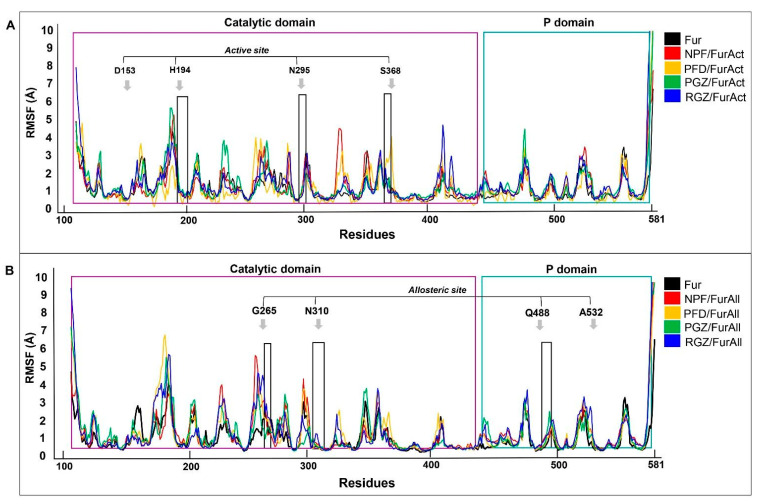
Root mean square fluctuation (RMSF) values of ligand/Fur complexes within the Fur active site (FurAct; (**A**)) and Fur allosteric site (FurAll; (**B**)) during MD simulation for 100 ns. NPF: naphthofluorescein; PFD: pirferidone, PGZ: pioglitazona, RGZ: rosiglitazone. Figures obtained with Carma v.2.01 and Excel software v. 2406. Arrows indicate the critical residues on the Fur active site. The purple box indicates the catalytic domain. The blue box indicates the P domain.

The complexes between the active site of Fur and NPF, PFD, PGZ, and RGZ showed a more significant number of unique fluctuating regions in contrast to the ligand-free Fur. In particular, the NPF/FurAct complex showed seven highly fluctuating regions located in the catalytic domain, with Regions 2 (Arg185, Tyr186, Gln188, Met189, and Asn190) and 5 (Phe323, Gly324, Asn325, Val326, and Pro327) being the most prominent, with distances of approximately 5.3 and 4 Å, respectively (Figure 7A).

The PFD/FurAct and RGZ/FurAct complexes also showed nine regions with high fluctuation, located mainly in the catalytic domain and, to a lesser extent, in the P domain (Figure 7A). Finally, the PGZ/FurAct complex showed the fewest regions of the fluctuating protein as the residue modifications, mainly in the catalytic domain, were smaller than for the other complexes; however, Region 2 (Pro184, Arg185, Tyr186, Thr187, Gln188, Met189, and Asn190) of this complex showed the most significant movement in its structure, reaching a peak of ~5.6 Å (Figure 7A).

In contrast to previous observations of FurAct, the FurAll complexes showed lower RMSF values and fewer fluctuating regions, comparable to that of ligand-free Fur. Similar to what we observed for the NPF/FurAll complex, only four highly fluctuating regions were observed compared to the Fur-alone protein, highlighting a region located in the P domain (Leu475 and Gly476) with an RMSF value of 4.15 Å (Figure 7B).

Also, the NPF/FurAll, PFD/FurAll, and RGZ/FurAll complexes showed five regions with high fluctuation with respect to the ligand-free Fur observed with FurAct. In both cases, the region comprising amino acids Asp258 to Val263 was the most variable, with an RMSF peak of ~5 A. The other regions identified in both complexes showed fluctuations ranging from 4 to 1.7 Å (Figure 7B).

Interestingly, the PGZ/FurAll complex’s fluctuating regions increased to eight when the ligand interacted with the Fur allosteric site. The regions with the greatest movement correspond to amino acids Thr187 to Met189 and Asp258 to Thr262, located near the Fur active site (Figure 7B).

The data obtained from RMSD, Rg, and RMSF suggest that the complexes formed with FurAll are more stable and compact than those directed to the FurAct, which could confirm that FurAll contains an important binding and control site of Fur and is adequate for binding with our ligands. Furthermore, when evaluating the ligands/Fur complexes’ specific interactions towards the active site in the last 10% of the MD simulation trajectories, we observed that all the ligands, except for NPF, remained in the determined site of interaction. Similarly, the MD simulations showed that all ligands respected their interaction site towards FurAll (Figures S4–S7). However, the amino acid residues involved in establishing the complexes at the end of the MD simulation of FurAct the FurAll sites differed from those observed by molecular docking (Figures S4–S7).

Despite the difference in amino acid residues involved in the formation of the complexes, all models, except the ligand-free Fur protein model, showed a similar conformation to that observed by molecular docking (Figure S8).

## 4. Discussion

Most of the existing Fur inhibitors are based on polypeptide molecules that resemble the native substrates of this enzyme, occupying the space within the catalytic site and inhibiting its function, as reported for peptidyl chloromethyl ketone (CMK), α1-antitrypsin variants, and meta-guanidinomethyl-Phac-RVR-Amba, among others [25,53,74,75]. Besides this, multiple low-molecular-weight non-peptide molecules may have an inhibitory capacity of Fur proteolytic activity, for example, the guanidinylated aryl 2,5-dideoxystreptamine derivatives (GADDs). However, these are not often used as therapeutic molecules due to their potential toxicity and lack of research efforts. Thus, the repositioning of therapeutic molecules (used for treating known conditions) represents an interesting and relatively rapid strategy to establish new therapeutic alternatives [37,40,76,77].

The use of non-peptide molecules as Fur inhibitors presents several advantages compared to peptide inhibitors, such as improved solubility, bioavailability, cell permeability, and metabolic and proteolytic stability. Some small non-peptide molecules that have shown inhibitory effects in Fur are labdane diterpenes, terpyridine/quinoline complexes of Zn^+2^/Cu^+2^ ions, dideoxy streptamine analogs, dicumarine derivates, flavonoids, and NPF analogs. Most prior studies of these molecules have been carried out in vitro, providing information about their efficacy or toxicity [17,78,79], but lack information on their interaction with their target protein. For these reasons, the in silico study of the molecular interactions of non-peptide molecules currently approved to treat different diseases is crucial for their rapid repositioning to treat other existing or emerging ailments. Specifically, the non-peptide NPF and PFD molecules have been reported for their inhibitory function of Fur in in vitro models of several ailments, such as cancer, inflammatory and metabolic diseases, idiopathic pulmonary fibrosis, viral and bacterial infections, and neurological diseases such as Alzheimer’s disease [80,81]. However, the mechanisms and molecular interactions conferring their inhibitory property have not been studied so far. The in silico approach used here assessed the molecular interactions of NPF and, for the first time, PFD with active (FurAct) and allosteric (FurAll) sites of Fur and calculated the affinity forces of the ligand/protein complexes. Our results show that both molecules have a high affinity for FurAct and even higher affinity for FurAll.

The FurAct site consists of a highly twisted β-sheet composed of seven parallel and one antiparallel β-strand, flanked by five adjacent and two peripheral α-helices and two β-hairpin loops, which regulate Fur protease activity, specifically through the surrounding negatively charged aspartic acid and glutamic acid residues (Asp258, Asp306, Asp154, Asp19, Glu236, Glu264, Glu257, Glu230, and Glu233) as well as the catalytic amino acid triad (Asp153, His194, and Ser368) [41,82,83,84]. Furthermore, it has been reported that besides the key role of the active site in the activation of Fur, in the P domain, there may exist an allosteric site (interdomain), which plays a crucial role in the stabilization and functions of this protein [2,82]. This interdomain connection is located between the eight-stranded β-sandwich of the P domain (Pβ5 and Pβ6) and the jelly-roll β-barrels of the catalytic domain (loops Cβ5-Calpha4, Cβ6-Cβ7, Cβ7-Cβ8, and Cβ11-Cβ12). It is highly conserved between proprotein/prohormone convertases (PC) and is believed to stabilize the catalytic domain [82]. A hydrophobic core and several polar–salt bridges mediate the property of this region. Also, the observation that these interactions shield it from solvent surface patches in this area [82] may be an ideal characteristic for interactions with other inhibitory molecules [52,74].

In a recent study, Feng et al. (2023) [74], using molecular docking, predicted the existence of a new potential allosteric site and determined its localization between the S8 region of the catalytic domain (aa121-435) and the P domain (aa444-576) of Fur. This localization corresponds with the amino acid residues observed in the present study (Gly265, Ans310, Gln488, and Ala532) during MD with FurAll, predicting the interaction of our four ligands with the potential allosteric site within the interdomain region. In this regard, Feng and Cols (2023) [75] showed that the activity of Fur decreased upon permethrin (tested inhibitor) binding in a dose-dependent way even though Fur´s Km did not, suggesting that this protein presents an alternative control region, which the authors named the allosteric site. The authors proposed this site as a non-competitive binding site that mediates the action of Fur. Thus, the molecular dockings carried out in the present study suggest that NPF and PFD presented higher affinity and preferential binding for FurAll than FurAct, possibly due to their non-competitive binding and the ease of forming polar bonds in the complexes.

In the case of the NPF/FurAll complex, we observed three polar bonds involving Gly265, Ans310, and Gln488 residues of FurAll but only one polar bond involving Asp154 of FurAct. Peiter et al. (2022) [81] reported similar results, where the amino acids Gln488 and Ala532 were involved in stabilizing the NPF/Fur complex, which may be related to the high inhibitory capacity of this drug.

On the other hand, for the PFD/FurAll complex, we observed that the main amino acid involved through polar bonds was Ans310, while for the PFD/FurAct complex, the interactions were mainly due to non-polar forces through Pi–sulfur, Pi–sigma, and amide–Pi stacked bonds and only one polar contact with Gly255, which makes the complex more unstable. Dahms et al. (2016) [85] reported that Gly255 was a key substrate recognition element.

Similar to the observations for NPF and PFD, our results showed a higher affinity for the two thiazolidinediones tested here (PGZ and RGZ) for FurAll than for FurAct structures. The molecular dockings calculated affinity forces of PGZ and RGZ with FurAct of −8.6 and −8.1 kcal/mol, respectively, whereas with FurAll, the affinities were −8.7 and −7.9 kcal/mol, respectively. The decrease in affinity forces may be due to the number and type of interactions, the distance between interactions, and the amino acid residues involved in forming these complexes.

The PGZ/FurAct and PGZ/FurAll complexes presented three and two polar bonds, respectively, which stabilized the ligand/protein interactions. The most significant differences in the calculated affinities in both complexes were the distances of the polar bonds, which were smaller for the FurAct (2.5, 2.5, and 2.6 Å) than for FurAll (2.28 and 2.7 Å). Likewise, the PGZ/FurAct complex presented many hydrophobic forces. Compared to PGZ/FurAll, hydrophobic forces were the main forces involved in these ligand/protein interactions. They may contribute to a lipophilic environment of the binding cavity, which would facilitate ligand/protein interactions [86,87].

PGZ/FurAct and PGZ/FurAll complexes showed high affinity forces. The number of polar bonds and the hydrophobic forces were higher for PGZ/FurAll (Ser311 and Gln488) than for PGZ/FurAct (Asp154). However, the amino acids that participate in H-bond formation and hydrophobic forces of the PGZ/FurAll complex promoted high stability due to their localization of Pβ6, Calpha4, and Cβ7, which could increase the stability of the catalytic site and the activity of Fur [82].

NFP, PFD, PGZ, and RGZ are molecules that contain heterocyclic systems in their structure; these are relevant in pharmacology because they can modulate properties at both the pharmacokinetic and pharmacodynamic levels [88] and add important properties, such as lipophilicity, interaction capacity, high polarity, and toxic effect. Specifically, TZDs have a heterocycle sulfur functional group, which promotes various pharmacological activities [89]. In addition, the presence of oxygen-based functional groups seems to be an important factor for the stability and high affinity in all complexes.

An MD simulation was then run to validate the molecular docking observations. We observed that in the last 10% of the MD trajectory, the average RMSD was lower in the complexes directed towards the FurAct than towards the FurAll site. PGZ/FurAll (4.66 Å) and NPF/FurAll (4.52 Å) complexes were the most stable during the trajectory compared to the complexes towards the catalytic site (FurAct), which were similar to that of the ligand-free Fur trajectory (3.98 Å). Similarly, the Rg and RMSF values of the PGZ/FurAll complex were 22.93 Å, comparable to the ligand-free protein (22.46 Å), with few fluctuating regions during complex formation. However, PFD and RGZ were more stable. They presented more compactness towards FurAct (RMSD: 4.21 ± 0.08 Å, Rg: 2.57 ± 0.05 vs. RMSD: 4.12 ± 0.06 Å, Rg: 22.65 ± 0. 06) than the allosteric site (FurAll), contrary to what was observed with NPF (RMSD: 4.67 ± 0.13 Å, Rg: 22.62 ± 0.09) and RGZ (RMSD: 4.77 Å, Rg: 22.77 ± 0.10) regarding the allosteric site and Fur alone (RMSD: 3.98 ± 0.07 Å, Rg: 22.45 ± 0.06). Thus, PGZ could be a candidate for a non-competitive Fur inhibitor. At the same time, RGZ could be a candidate for a competitive and non-competitive Fur inhibitor for stably interacting with both the FurAct and the FurAll sites.

Feng et al. (2023) [75] highlighted the significance of the potential allosteric site of Fur for the design and repositioning of specific inhibitors of Fur, and other authors have also suggested the feasibility of inhibiting Fur through non-competitive means by allosteric binding of drugs [81,90]. Sheybani et al. (2021) [90] demonstrated that folic acid and folinic acid bind Fur through an H-bond with amino acid residues that belong to the allosteric site (interdomain region) (folic acid: Gly307, Glu271, Tyr313, Gln488, Ala532, Arg490, and Asp530; phenolic acid: Ser311, Glu271, Arg490, Lys449, and Gln488). On the other hand, Peiter et al. (2022) [90] showed that NPF might inhibit Fur by binding Ala532 and Gln488.

The molecular docking data presented here showed slight differences between the AutoDock4 v.4.2.6 and AutoDock Vina software v.2.5.4. This is most probably due to the properties of each program, as explained by Nguyen et al. (2020) [91], who mentioned that AutoDock Vina better illustrates the binding and poses of the ligands with the protein of interest, while AutoDock4 is more accurate in the calculation of the binding affinity between the ligand and the protein. However, molecular docking assays with both programs showed interaction patterns similar to those observed in MD.

According to the molecular interactions, affinity, and stability of complexes, the Ki values calculated by AutoDock4 were consistent with the calculated affinity forces and showed low Ki values for the four molecules studied here, which is related to their high-affinity forces towards their specific interaction sites. PFD was better against FurAct compared to NFP, PGZ, and RGZ, in which Ki values were very low against FurAll and coincided with a high-affinity force of each specific molecule for these sites. 

Ki is the inhibitor concentration required to decrease the enzyme’s reaction by half. Therefore, a lower Ki is associated with low or poor dissociation of the inhibitors from the target, i.e., the inhibition is more stable [92].

Therefore, the TZD studied here might have the capacity to bind to Fur as well as NPF and PFD. However, their mechanism of action may not only involve competitive inhibition through binding to the active site (as NPF and PFD do) but also a non-competitive inhibition through destabilization of the interdomain region (catalytic domain and P domain) of Fur. This function of the TZD will be further explored as an antiviral, a function recently observed for NPF against SARS-CoV2 by Cheng et al. (2020) [37]. Further studies are needed to scrutinize this point, as Fur convertase activity plays a key role in the entry of the virus into its host cells during the infectious process [5,49].

Some limitations should be considered for this study. While molecular dynamics simulations provide a valuable starting point for understanding how molecules interact with proteins and assessing their stability, additional in silico analyses like Poisson–Boltzmann, generalized Born, and surface area continuum solvation give more information regarding the union stability. Moreover, enzymatic assays further refine this understanding by confirming both stability and affinity. Future studies should investigate whether these effects hold true under more challenging conditions, as such molecules could offer promising leads for drug development.

## 5. Conclusions

In conclusion, this study employed in silico methods to identify PGZ and RGZ, which belong to the TZD group, as potential inhibitors of furin. Notably, these molecules have not been previously explored for this purpose. Our analysis revealed favorable binding interactions between these novel inhibitors and furin, similar to those observed with established inhibitors like NPF and PFD. These findings provide a valuable starting point for further research. Experimental testing is now crucial to validate the inhibitory potential of TZD against furin. Additionally, the insights gained regarding NPF and PFD binding modes bring new knowledge for their optimization as furin inhibitors. The potential for repurposing existing drugs like PGZ and RGZ, already tested for safety and side effects in type 2 diabetes patients, could significantly accelerate the development of new therapies. This approach holds promise for diseases with highly unmet medical needs or emerging infectious diseases like SARS-CoV-2. Further research is now necessary to determine their efficacy and safety for specific applications.

## Figures and Tables

**Table 1:** Zinc15 database IDs of the 3D structures of four potential Fur ligands: NPF, PFD, PGZ, and RGZ.

ID	Name and Abbreviation	Structure
PubChem CID 3124834	Naphthofluorescein NPF	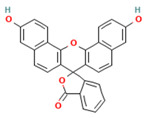
ZINC1958	Pirfenidone PFD	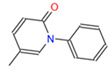
ZINC968326	Pioglitazone PGZ	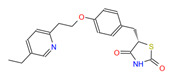
ZINC968328	Rosiglitazone RGZ	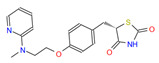

NPF: naphtofluorescein; PFD: pirfenidone; PGZ: pioglitazone; RGZ: rosiglitazone.

**Table 2:** Summary of NPF, PFD, PGZ, and RGZ molecular interactions with FurAct by localized molecular docking.

Ligands	Site Interaction	Free Energy of Binding (kcal/mol) AutoDock4	Free Energy of Binding (kcal/mol) Vina	H-Bond Classical and Non-Classical	Distance H-Bond (Å)	Electrostatic	Hydrophobic
Naphthofluorescein (NPF)	Active	NA (NA)	−9.9 (0.00)	Asp154—H Asp191—H	2.7 3.1	Asp258	Val231 Leu227
Pirfenidone (PFD)	Active	−6.28 (0.00)	−6.9 (1.709)	Gly255—O	3.4	Glu236	Gly255 Trp254 Trp291 Leu227 Tyr308 Val231 Pro256
Pioglitazone (PGZ)	Active	−8.04 (0.00)	−8.6 (0.00)	Asp154—H Asp191—H Asn192—O	2.6 2.5 2.6	NA	Leu227 Val231 Leu152 Met226 Trp254 Leu240 Tyr308 Trp291
Rosiglitazone (RGZ)	Active	−7.47 (0.00)	−8.1 (0.00)	Asp154—H	2.2	NA	Leu227 Val231 Met226 Trp291 Ala252 Tyr308

NA: not available; Å = angstrom; NPF: naphthofluorescein; PFD = pirfenidone; PGZ: pioglitazone; RGZ: rosiglitazone.

**Table 3:** Summary of molecular interactions of NPF, PFD, PGZ, and RGZ with FurAll by blind docking.

Ligands	Site Interaction	Free Energy of Binding (kcal/mol) Blind Docking Server	H-Bond Classical and Non-Classical	Distance H-Bond (Å)	Electrostatic	Hydrophobic
Naphthofluorescein (NPF)	Allosteric	−10.9	Gln488—H Asn310—O Gly265—O	2.3 2.4 2.3	NA	Pro266 Trp531 Ser311 Ala532 Glu271
Pirfenidone (PFD)	Allosteric	−7.1	Asn310—O	2.6	Glu271	Ala532 Trp531 Tyr313 Ile312
Pioglitazone (PGZ)	Allosteric	−8.7	Ser311—H Gln488—O	2.2 2.7	NA	Trp531 Val263 Ile312
Rosiglitazone (RGZ)	Allosteric	−7.9	Asn310—H Ser311—O Val263—N Glu271—O	1.9 2.2 3.3 3.4	NA	Trp531 Val263 Asp264 Gly307

NA: not available; Å = angstrom; NPF: naphthofluorescein; PFD = pirfenidone; PGZ: pioglitazone; RGZ: rosiglitazone.

**Table 4:** Inhibition constant (Ki) of Fur by NFP, PFD, PGZ, and RGZ.

	Inhibition Constant (Ki)	
Ligands	Localized Docking (FurAct)	Blind Docking (FurAll)
NFP	NA	201.04 nM
PFD	24.87 μM	NA
PGZ	1.28 μM	970.36 nM
RGZ	3.33 μM	1.49 μM

NA: not available. NFP: naphtofluorescein; PFD: pirfenidone; PGZ: pioglitazone; RGZ: rosiglitazone.

**Table 5:** Average RMSD of the ligands/FurAct and ligands/FurAll complexes during the last 10% of the molecular dynamics.

Complexes	RMSD (Å) of Active Site (100 ns)	RMSD (Å) of Allosteric Site (100 ns)
Ligand-free Fur	3.98 ± 0.07	3.98 ± 0.07
NPF/Fur	4.67 ± 0.13	4.52 ± 0.05
PFD/Fur	4.21 ± 0.08	5.22 ± 0.17
PGZ/Fur	4.12 ± 0.06	4.66 ± 0.17
RGZ/Fur	4.77 ± 0.14	5.57 ± 0.17

NPF: naphtofluorescein; PFD: pirfenidone; PGZ: pioglitazone; RGZ: rosiglitazone.

**Table 6:** Average Rg of ligand/Fur complexes between the Fur active and allosteric sites during the last 10% of the molecular dynamics.

Complexes	Rg (Å) of Active Site (100 ns)	Rg (Å) of Allosteric Site (100 ns)
Ligand-free Fur	22.45 ± 0.06	22.4 ± 0.06
NPF/Fur	22.62 ± 0.09	22.56 ± 0.06
PFD/Fur	22.57 ± 0.05	22.90 ± 0.06
PGZ/Fur	22.65 ± 0.06	22.93 ± 0.06
RGZ/Fur	22.77 ± 0.10	23.22 ± 0.06

NPF: naphtofluorescein; PFD: pirfenidone; PGZ: pioglitazone; RGZ: rosiglitazone.

## Data Availability

Data are contained within the article and Supplementary Materials. Further inquiries can be directed to the corresponding authors.

## Supplementary Materials

The following supporting information can be downloaded at https://www.mdpi.com/article/10.3390/cimb46080511/s1: Figure S1: The 3D representation of redocking of crystallographic structures and co-crystalized ligands. (A) 7QXZ structure with -(3,5-dichlorophenyl) pyridine-derived inhibitor, (B) 5MIM structure with 2,5-dideoxystreptamine-derived inhibitor, (C) 7O1Y with guanylhydrazone-based inhibitor 2 (mi307), and (D) 7LCU structure with BOS-318 inhibitor. Figures obtained with PyMOL v.2.5.4. Blue-green color represents the crystallographic structures with its ligand inhibitors, and pink represents structure redocking with its ligand inhibitors; Figure S2: Representation of the 3D and 2D structures of the molecular interactions between the Fur active site (FurAct) and 3-(3,5-dichlorophenyl) pyridine-derived (3DPP-D) inhibitor. (A) Full complex, (B) specific interactions of 3DPP-D. Figures obtained with the PyMOL v.2.5.4 and Discovery Studio v.21.1.0.20298 software; Figure S3: The 3D and 2D representations of the Fur active site (FurAct) and its molecular interactions with the potential ligands. (A,B) Overview of overlay complexes, (C) NPF, (D) PFD, (E) PGZ, and (F) RGZ. Figures obtained with the PyMOL v.2.5.4 and Discovery Studio v.21.1.0.20298 software; Figure S4: The 3D representation of the models of the complexes between FurAct and its potential ligands during the last 10% of molecular dynamics simulation. (A) NPF (naphtofluorescein), (B) PFD (pirfenidone), (C) PGZ (pioglitazone), and (D) RGZ (rosiglitazone). Figures obtained with PyMOL2; Figure S5: The 2D representation of models of the complex formed between FurAct and its potential ligands in the last 10% of molecular dynamics simulation. (A) NPF (naphtofluorescein), (B) PFD (pirfenidone), (C) PGZ (pioglitazone), and (D) RGZ (rosiglitazone). Figures obtained with the PyMOL v.2.5.4 and LigPlot+ v.2.2.8 software; Figure S6: The 3D representation of models of the complex between FurAll and its potential ligands during the last 10% of molecular dynamics simulation. (A) NPF (naphtofluorescein), (B) PFD (pirfenidone), (C) PGZ (pioglitazone), and (D) RGZ (rosiglitazone). Figures obtained with PyMOL v.2.5.4; Figure S7: The 2D representation of models of the complex formed between FurAll and its potential ligands in the last 10% of molecular dynamics simulation. (A) NPF (naphtofluorescein), (B) PFD (pirfenidone), (C) PGZ (pioglitazone), and (D) RGZ (rosiglitazone). Figures obtained with the PyMOL v.2.5.4 and LigPlot+ v.2.2.8 software; Figure S8: Comparison of the 3D representation of the complex formed between FurAct and FurAll and the potential ligands obtained by molecular docking and molecular dynamics simulation. Figures obtained with Discovery Studio v.2021. Yellow boxes represent the interaction site determined by molecular dockings, and green boxes represent the interaction site determined by molecular dynamics; Table S1: Redocking of structures of furin with their co-crystalized ligands; Table S2: Summary of the molecular interactions of 3DPP-D with FurAct and FurAll; Table S3: Summary of the molecular interactions of NPF, PFD, PGZ, and RGZ with FurAll by general molecular docking.
