# Supplementary material for: Evaluation of Potential Furin Protease Inhibitory Properties of Pioglitazone, Rosiglitazone, and Pirfenidone: An In Silico Docking and Molecular Dynamics Simulation Approach
Source: Curr Issues Mol Biol. 2024 Aug 8;46(8):8665–84. doi: 10.3390/cimb46080511 (PMC11352479; doi:10.3390/cimb46080511)
Supplement: Supplementary file 1 [file cimb-46-00511-s001.zip › Supplementary Figures.pdf]

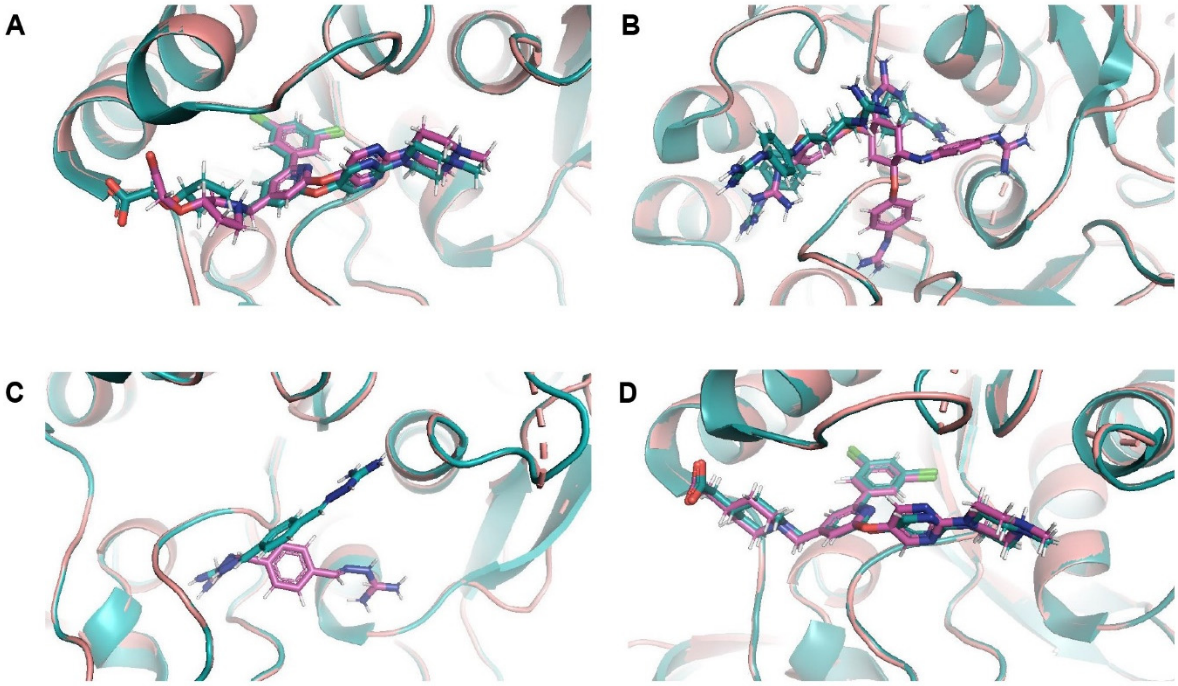


**Figure S1.** 3D representation of redocking of crystallographic structures and its co-cristalizated lignads. A) 7QXZ structure with -(3,5-Dichlorophenyl) pyridine-derived, B) 5MIM structure with 2,5-dideoxystreptamine derived, C) 7O1Y with guanylhydrazone-based inhibitor 2 (mi307), D) 7LCU structure with BOS-318 inhibitor. (Figures obtained with PyMOL v. 2.5.4). Blue-green color represent the crystallographic structures with its ligand inhibitors and pink structure represent structure redocking with its ligand inhibitors.

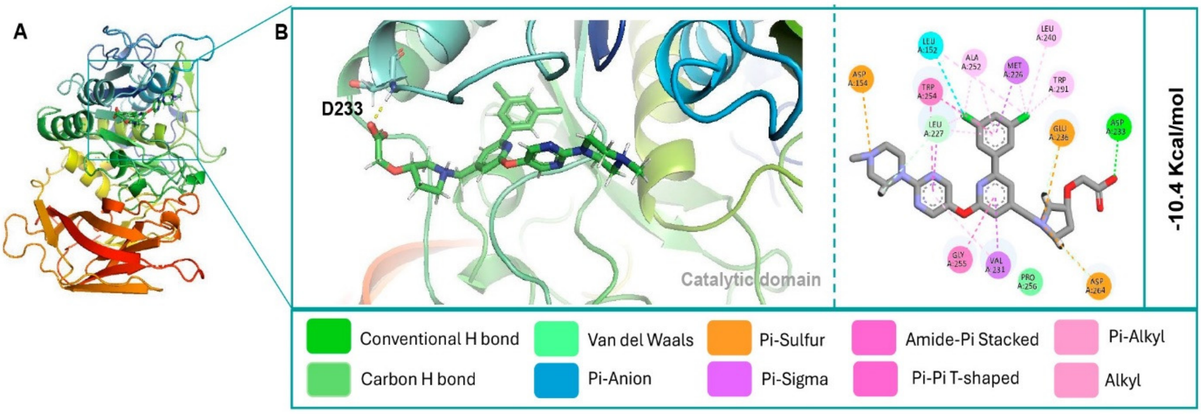


**Figure S2.** Representation of the 3D and 2D structures of the molecular interactions between the Fur active site (FurAct) and 3-(3,5-Dichlorophenyl) pyridine-derived (3DPP-D). A) Full complex, B) Specific interactions of 3DPP-D. (Figures obtained with the PyMOL v. 2.5.4 and Discovery studio v.21.1.0.20298 softwares).

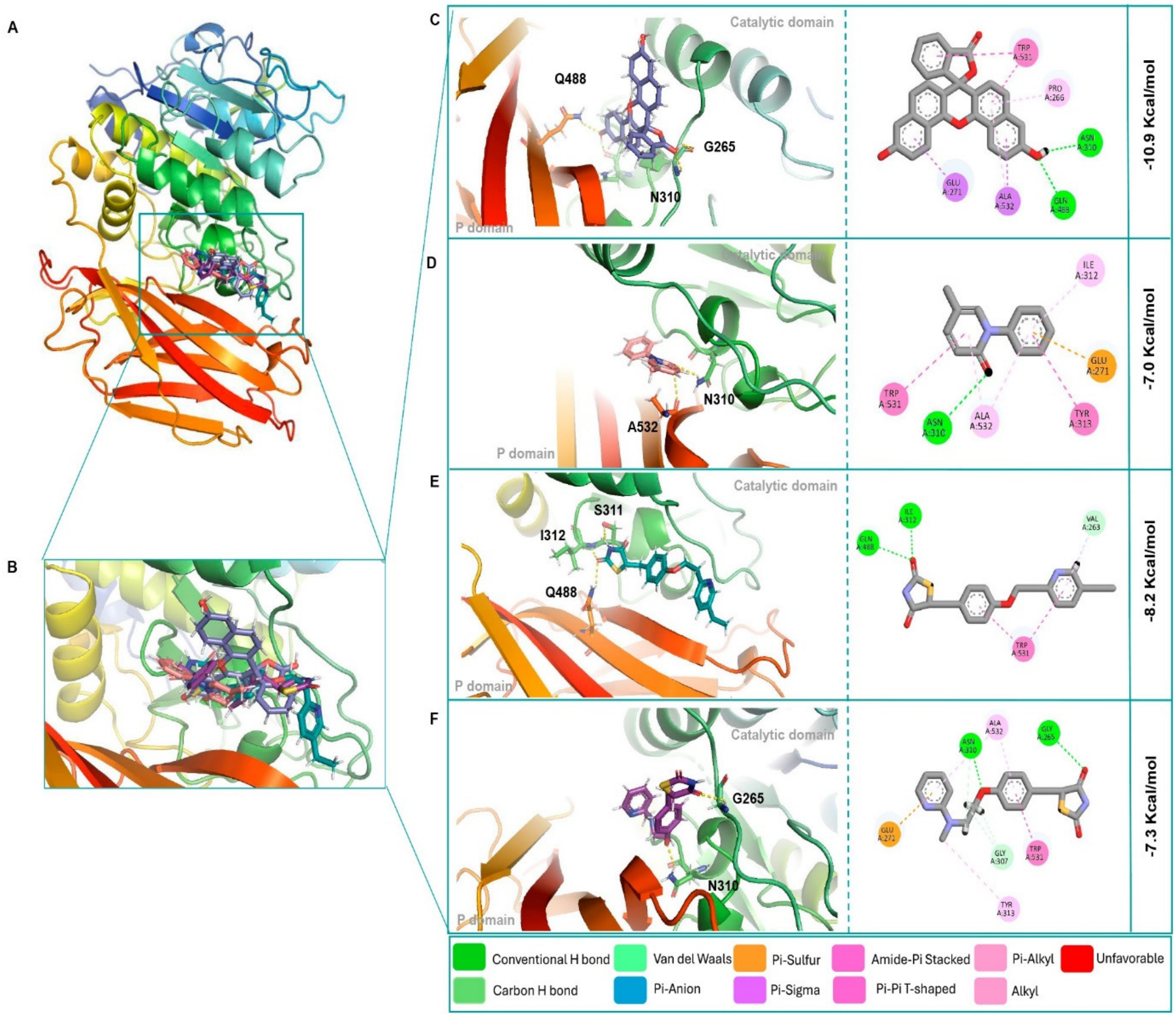


**Figure S3.** 3D and 2D representations of Fur active site (FurAct) and its molecular interactions with the potential ligands A-B) Overview of overlay complexes, C) NPF, D) PFD, E) PGZ, F) RGZ. (Figures obtained with the PyMOL v. 2.5.4 and Discovery studio v.21.1.0.20298 softwares).

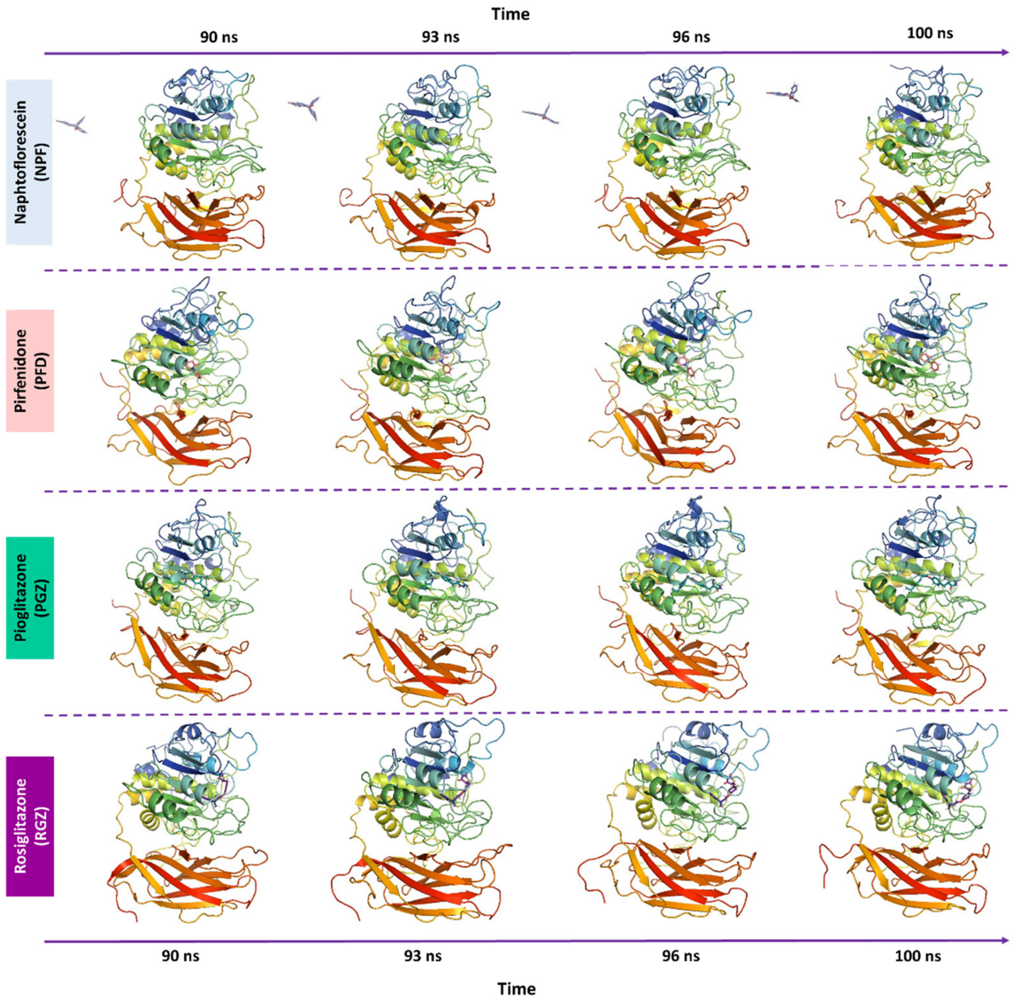


**Figure S4**. 3D representation of the models of the complexes between FuAct and its potential ligands during the last ten percent of molecular dynamic simulation. A) NPF (Naphtofluorescein), B) PFD (pirfenidone), C) PGZ (pioglitazone), D) RGZ (rosiglitazone). (Figures obtained with PyMOL2).

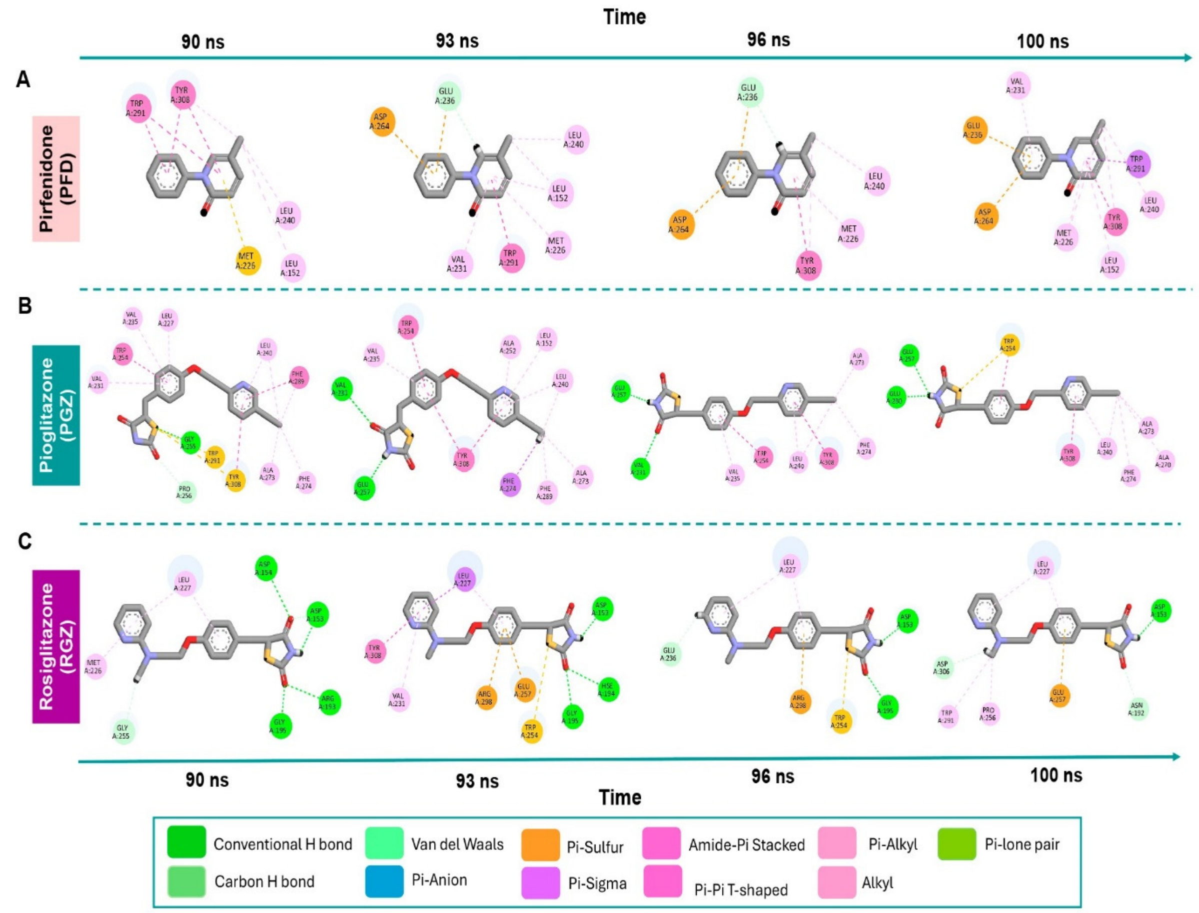


**Figure S5**. 1. 2D representation of models of the complexes formed between FuAct and its potential ligands in the last ten percent of molecular dynamic simulation. A) NPF (Naphtofluorescein), B) PFD (pirfenidone), C) PGZ (pioglitazone), D) RGZ (rosiglitazone). (Figures obtained with the PyMOL v. 2.5.4 and LigPlot+ v.2.2.8 softwares).

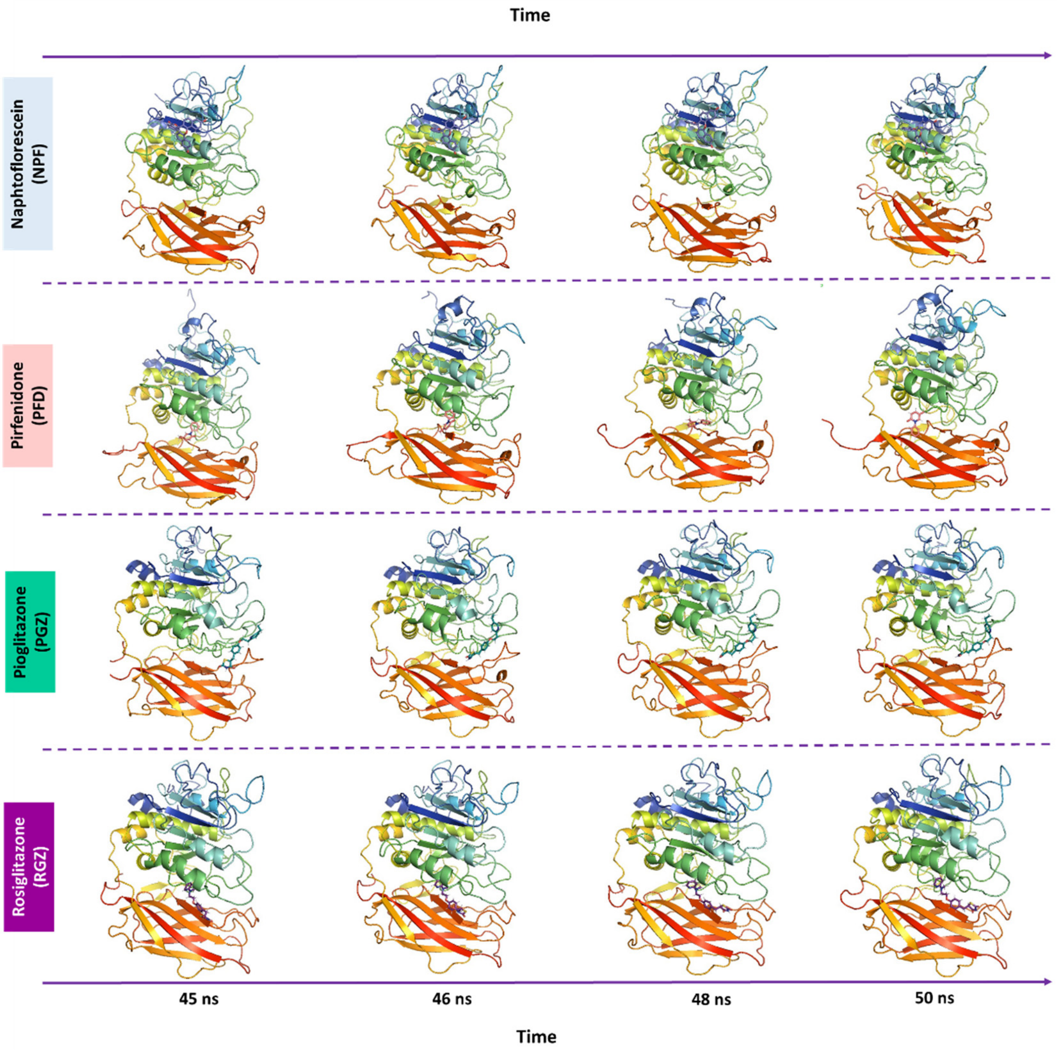


**Figure S6.** 3D representation of models of the complex between FuAll and its potential ligands during the last ten percent of molecular dynamic simulation. A) NPF (Naphtofluorescein), B) PFD (pirfenidone), C) PGZ (pioglitazone), D) RGZ (rosiglitazone). (Figures obtained with PyMOL v. 2.5.4).

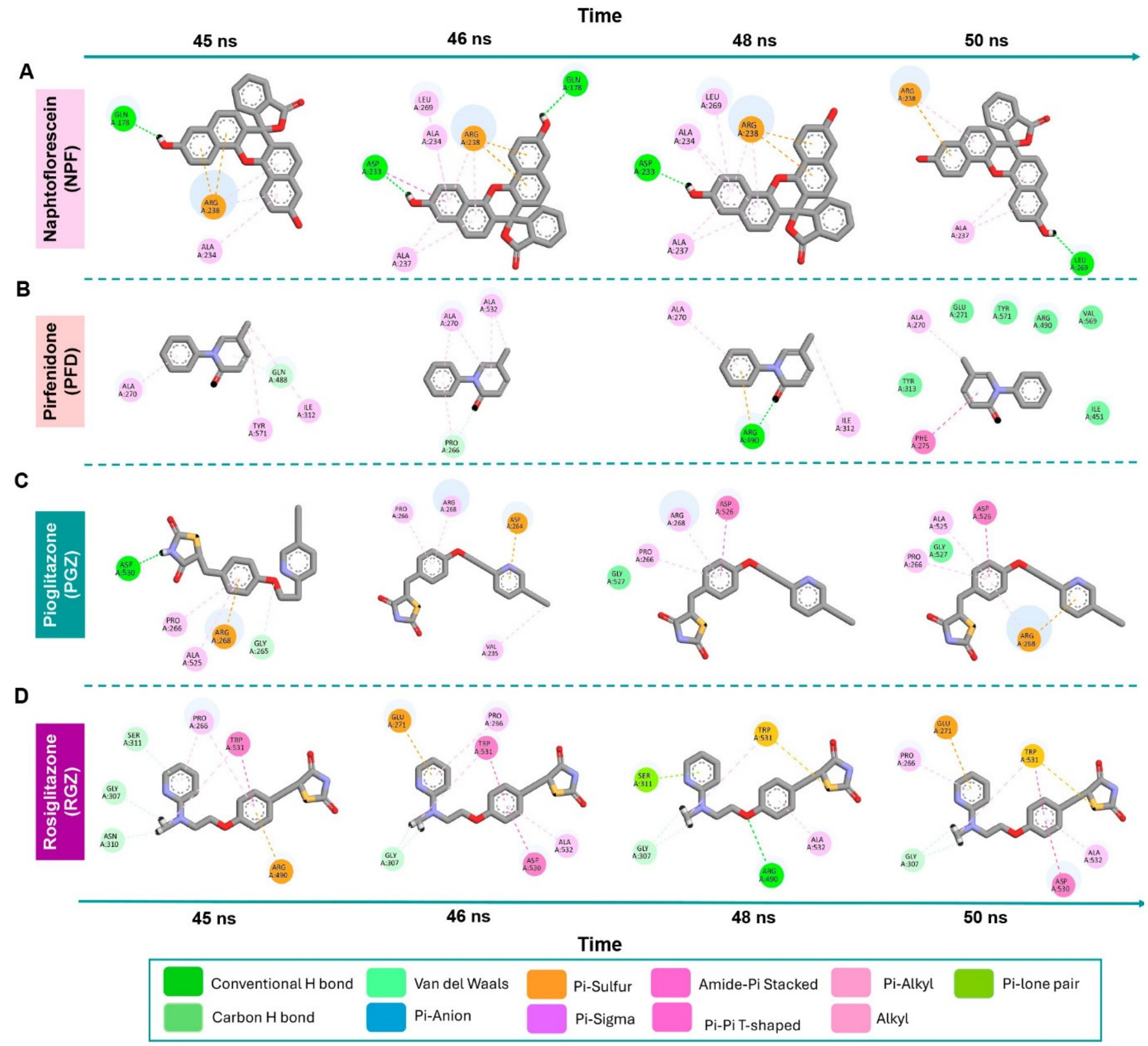


**Figure S7.** 2D representation of models of the complexes formed between FuAll and its potential ligands in the last ten percent of molecular dynamic simulation. A) NPF (Naphtofluorescein), B) PFD (pirfenidone), C) PGZ (pioglitazone), D) RGZ (rosiglitazone). (Figures obtained with the PyMOL v. 2.5.4 and LigPlot+ v.2.2.8 softwares).

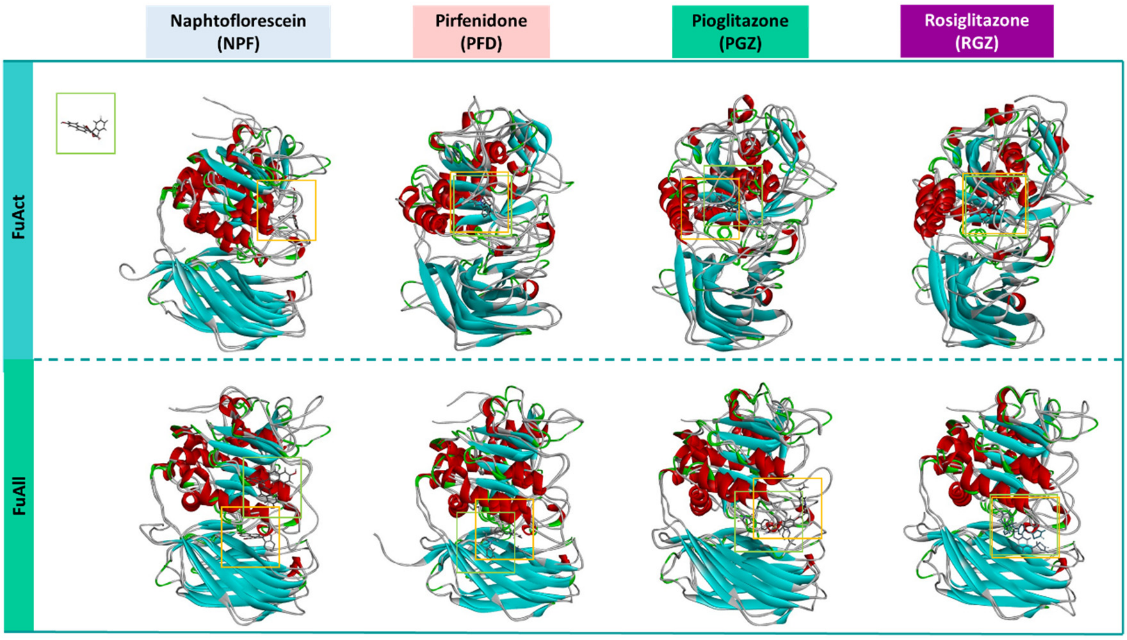


**Figure S8.** Comparison of 3D representation of the complexes formed between FuAct and FuAll and the potential ligands obtained by molecular docking and molecular dynamic simulation. (Figures obtained with Discovery Studio V. 2021). Yellow boxes represent the interaction site determined by molecular dockings, and green boxes represent the interaction site determined by molecular dynamics.
