# Supplementary material for: Evaluation of Potential Furin Protease Inhibitory Properties of Pioglitazone, Rosiglitazone, and Pirfenidone: An In Silico Docking and Molecular Dynamics Simulation Approach
Source: Curr Issues Mol Biol. 2024 Aug 8;46(8):8665–84. doi: 10.3390/cimb46080511 (PMC11352479; doi:10.3390/cimb46080511)
Supplement: Supplementary file 1 [file cimb-46-00511-s001.zip › Table S1.pdf]

Table S1. Redocking of strucutres of Furin with their ligands co-cristalizated.

Structure Furin (PDB ID)	Resolution (Å)	Ligands	Free Energy of Binding (Kcal/mol) VINA	RMSD	Free Energy of Binding (Kcal/mol) Autodock 4.2	RMSD
7QXZ	1.8	3-(3,5-Dichlorophenyl) pyridine-derived	-10.4	1.444	-14.16	1.05
5MIM	1.9	2,5-dideoxystreptamine derived	-7.8	2.104	-7.31	2.61
7LCU	1.34	BOS-318	-10.3	0.00	-13.98	1.13
71OY	1.7	Guanylhydrazone-based inhibitor 2	-6.1	3.37	-6.6	2.31
