# Supplementary material for: Evaluation of Potential Furin Protease Inhibitory Properties of Pioglitazone, Rosiglitazone, and Pirfenidone: An In Silico Docking and Molecular Dynamics Simulation Approach
Source: Curr Issues Mol Biol. 2024 Aug 8;46(8):8665–84. doi: 10.3390/cimb46080511 (PMC11352479; doi:10.3390/cimb46080511)
Supplement: Supplementary file 1 [file cimb-46-00511-s001.zip › Table S2.pdf]

**Table S2.** Summary of the molecular interactions of 3DPP-D with FurAct and FurAll.

| Ligands | Site interaction | Free Energy of Binding (Kcal/mol) and RMSD AUTODOCK4 | Free Energy of Binding (Kcal/mol) and RMSD VINA | H bond classical and non-classical | Distance H-bond (Å) | Electrostatic | Hydrophobic |
|---|---|---|---|---|---|---|---|
| 3-(3,5-Dichlorophenyl) pyridine-derived 3DPP-D | Active | -13.82 (0.98) | -10.2 (0.00) | Asp 233 – O | 2.1 | Pro 256 Asp 264 Glu 236 Asp 154 | Trp 254 Ala 252 Met 226 Leu 240 Trp 291 Val 231 Gly 255 |
| | Allosteric | NA (NA) | NA (NA) | NA | NA | NA | NA |
