# Supplementary material for: Evaluation of Potential Furin Protease Inhibitory Properties of Pioglitazone, Rosiglitazone, and Pirfenidone: An In Silico Docking and Molecular Dynamics Simulation Approach
Source: Curr Issues Mol Biol. 2024 Aug 8;46(8):8665–84. doi: 10.3390/cimb46080511 (PMC11352479; doi:10.3390/cimb46080511)
Supplement: Supplementary file 1 [file cimb-46-00511-s001.zip › Table S3.pdf]

Table S3. Summary of the molecular interactions of NPF, PFD, PGZ, and RGZ with FurAll by general molecular docking.

**Ligands**	**Site interaction**	**Free Energy of Binding (Kcal/mol) AUTODOCK4**	**Free Energy of Binding (Kcal/mol) VINA**	**H bond classical and non-classical**	**Distance H-bond (Å)**	**Electrostatic**	**Hydrophobic**
Naphthofluorescein (NPF)	Allosteric	-9.14 (0.00)	-10.9 (0.00)	Gly265 – O<br>Ans310 – H<br>Glu488 – O	2.4<br>2.1<br>2.3		Glu271<br>Ala532<br>Trp531<br>Pro266
Pirfenidone (PFD)	Allosteric	-6.28 (0.00)	-7.0 (0.00)	Ans310 – O<br>Ala532 – O	2.7<br>3.4	Glu271	Trp531<br>Ala532<br>Tyr313<br>Ile312
Pioglitazone (PGZ)	Allosteric	-8.20 (0.00)	-8.2 (0.00)	Ser311 – H<br>Ile312 – O<br>Gln488 – O	2.8<br>2.0<br>2.6	NA	Trp531
Rosiglitazone (RGZ)	Allosteric	-7.95 (0.00)	-7.3 (0.00)	Asn310 – O<br>Gly265 – O	2.1<br>2.4		Glu271<br>Ala532<br>Trp531<br>Pro266

NA= Not apply; = Ångstrom; NPF: Naphthofluorescein; PFD= Pirfenidone; PGZ: Pioglitazone; RGZ: Rosiglitazone.
